# Supplementary material for: Transcranial direct current stimulation modulates primate brain dynamics across states of consciousness
Source: eLife. 2025 Oct 13;13:RP101688. doi: 10.7554/eLife.101688 (PMC12517689; doi:10.7554/eLife.101688)

Monkey R. (8 MRI sessions)

Anodal electrode (prefrontal)

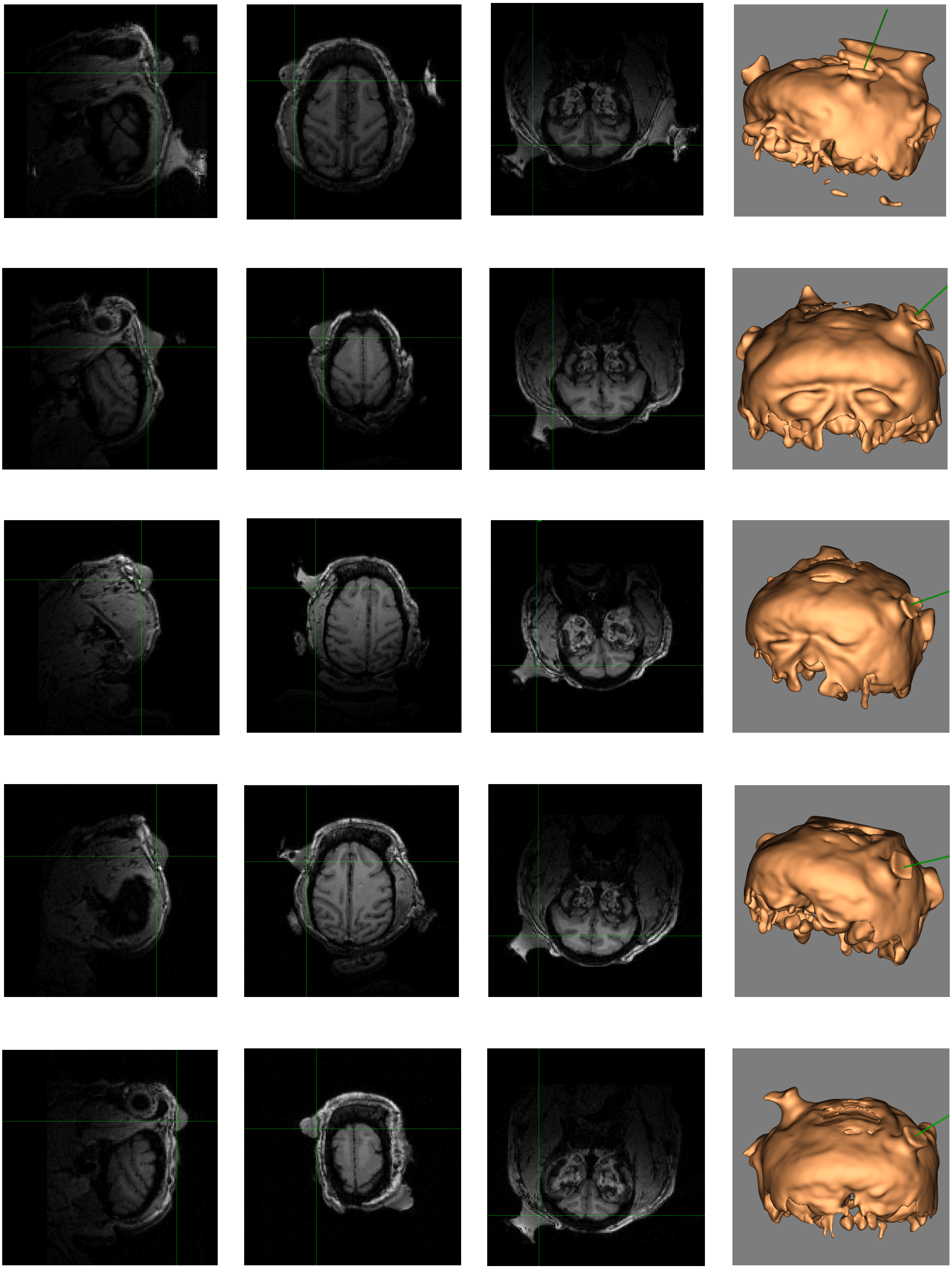

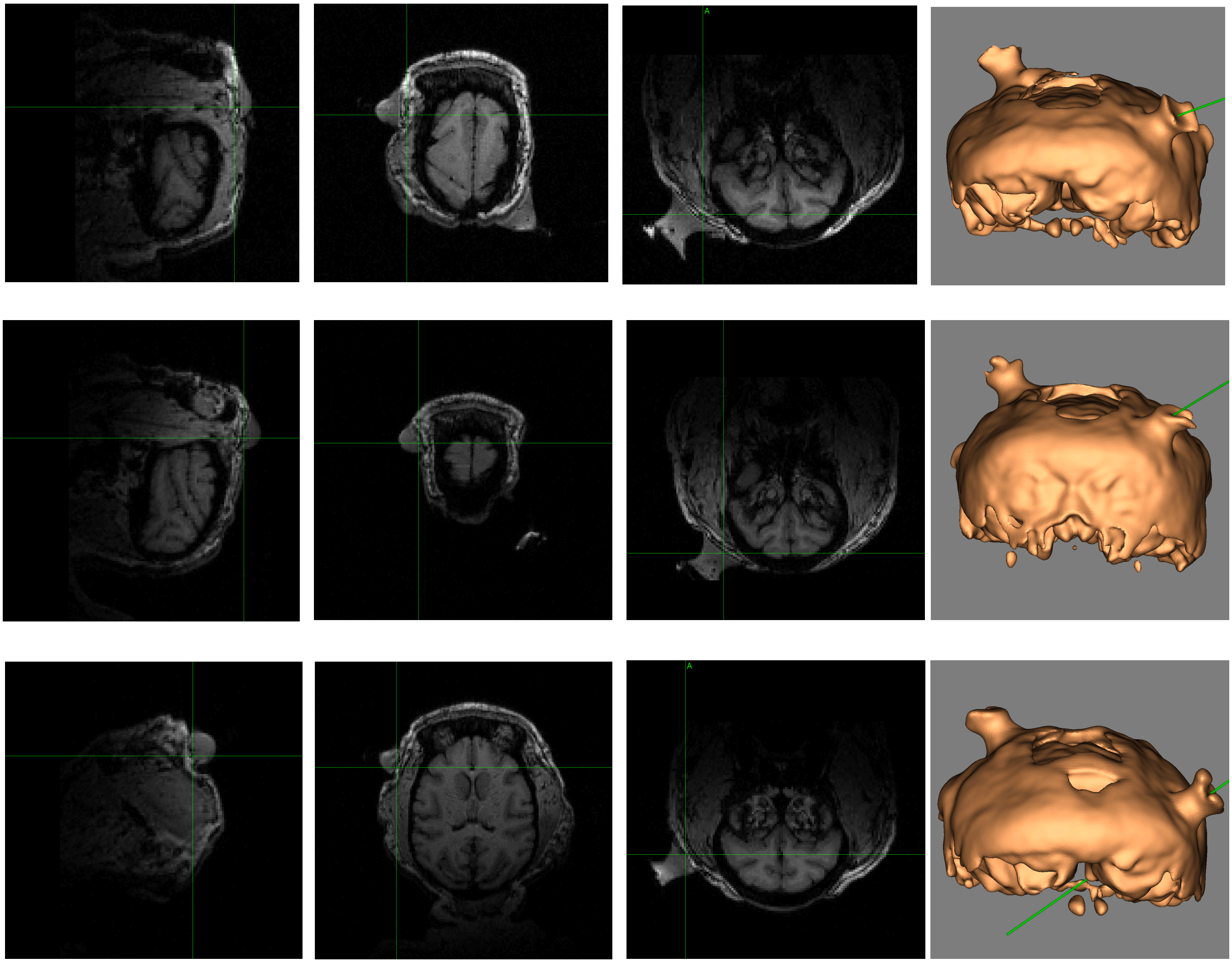

Cathodal electrode (occipital)

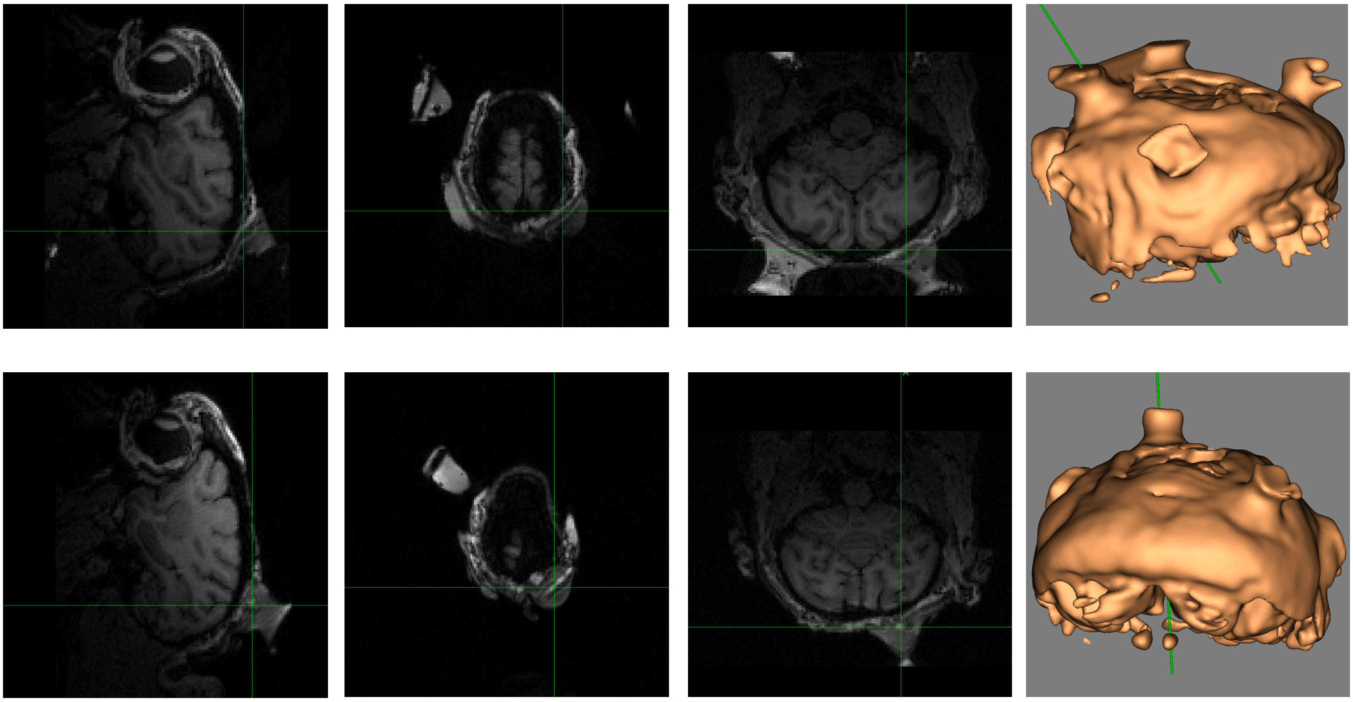

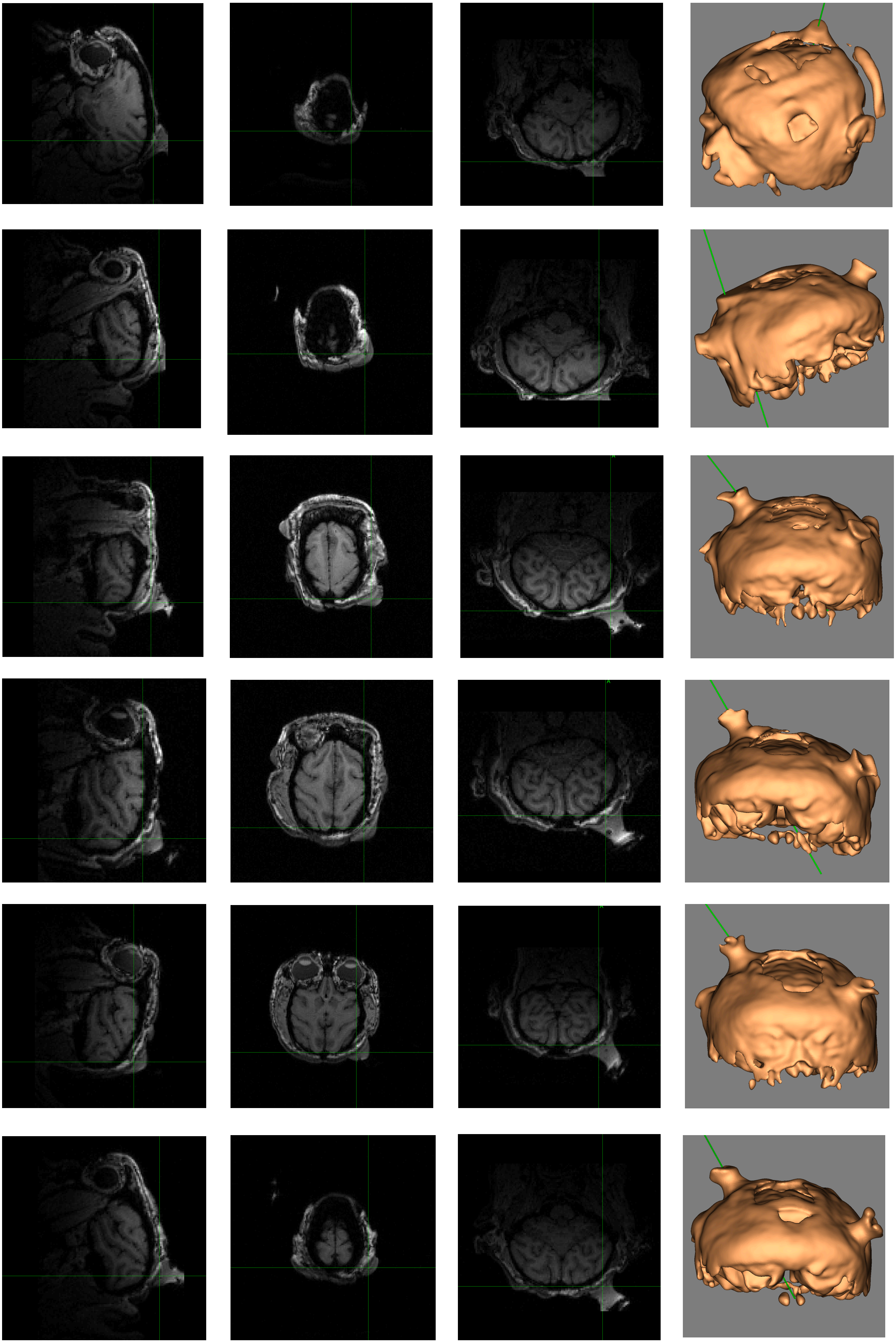

Monkey N. (2 MRI sessions)

Anodal electrode (prefrontal)

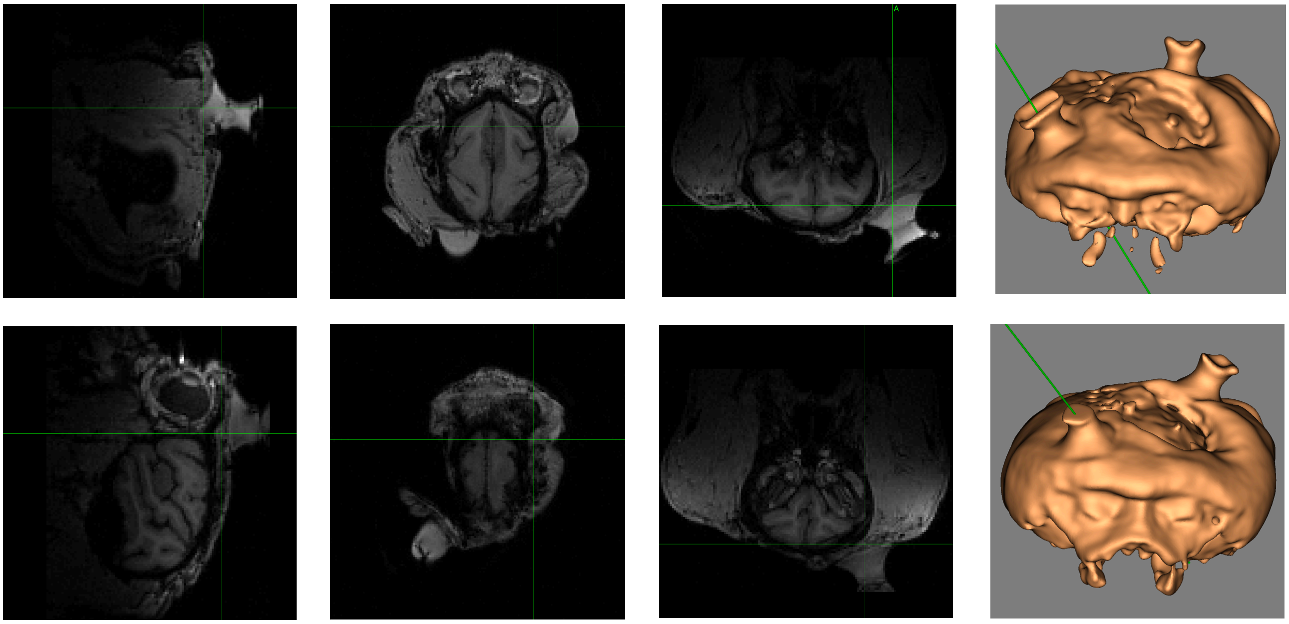

Cathodal electrode (occipital)

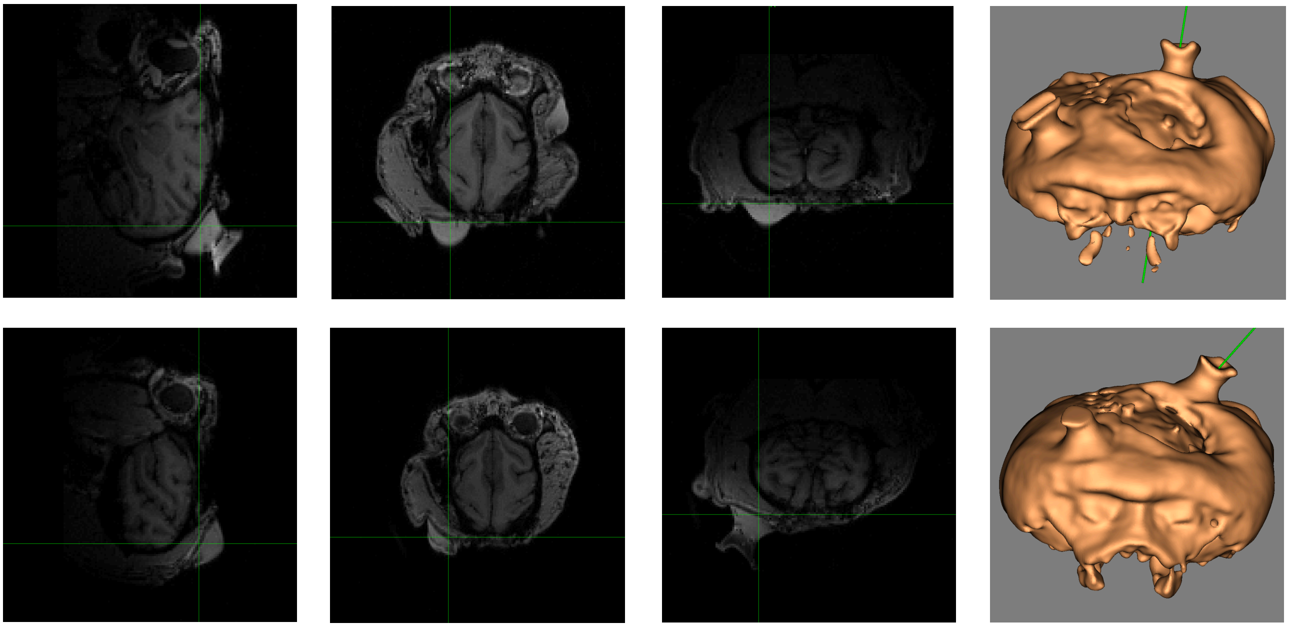

Monkey J. (16 MRI sessions)

Anodal electrode (prefrontal)

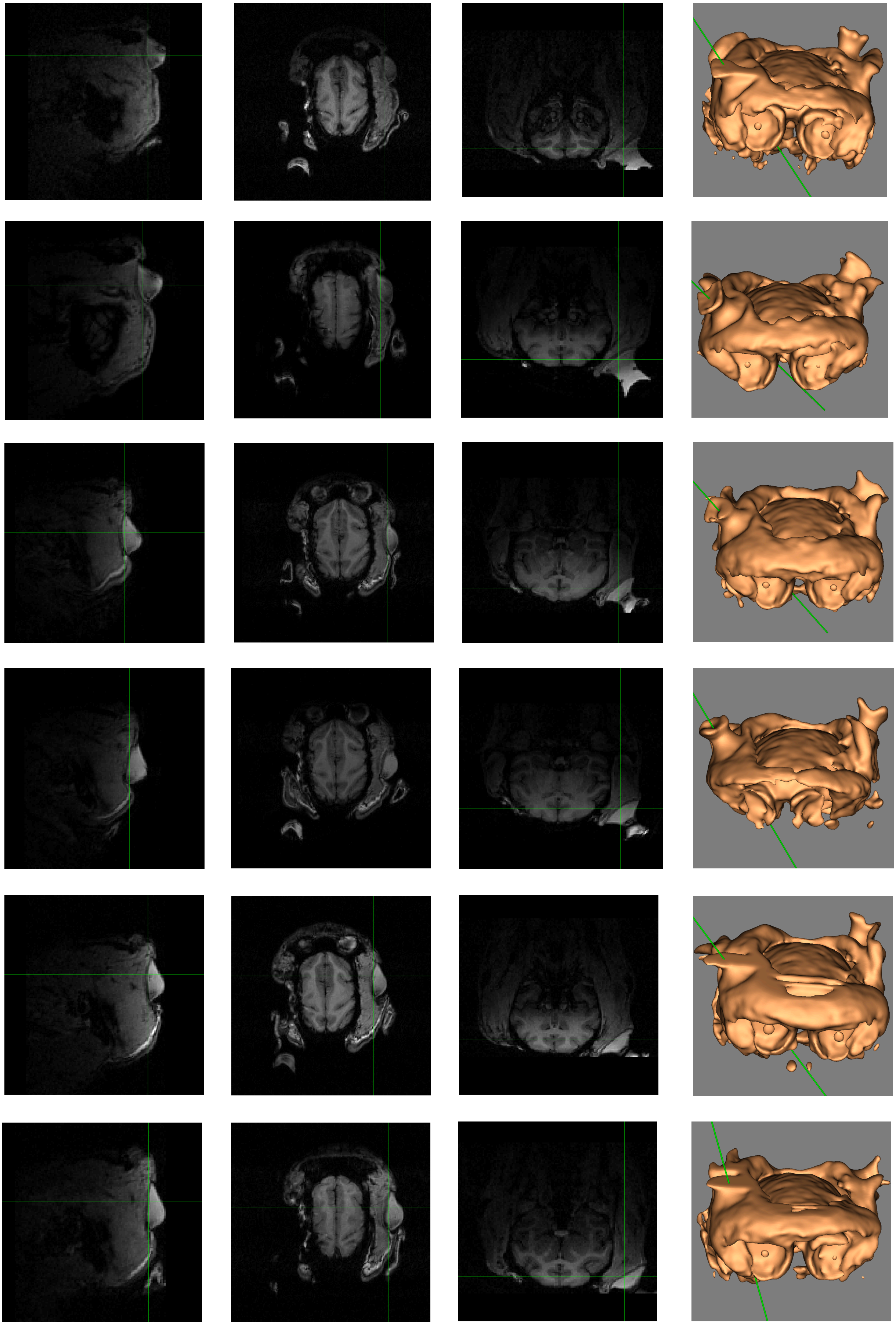

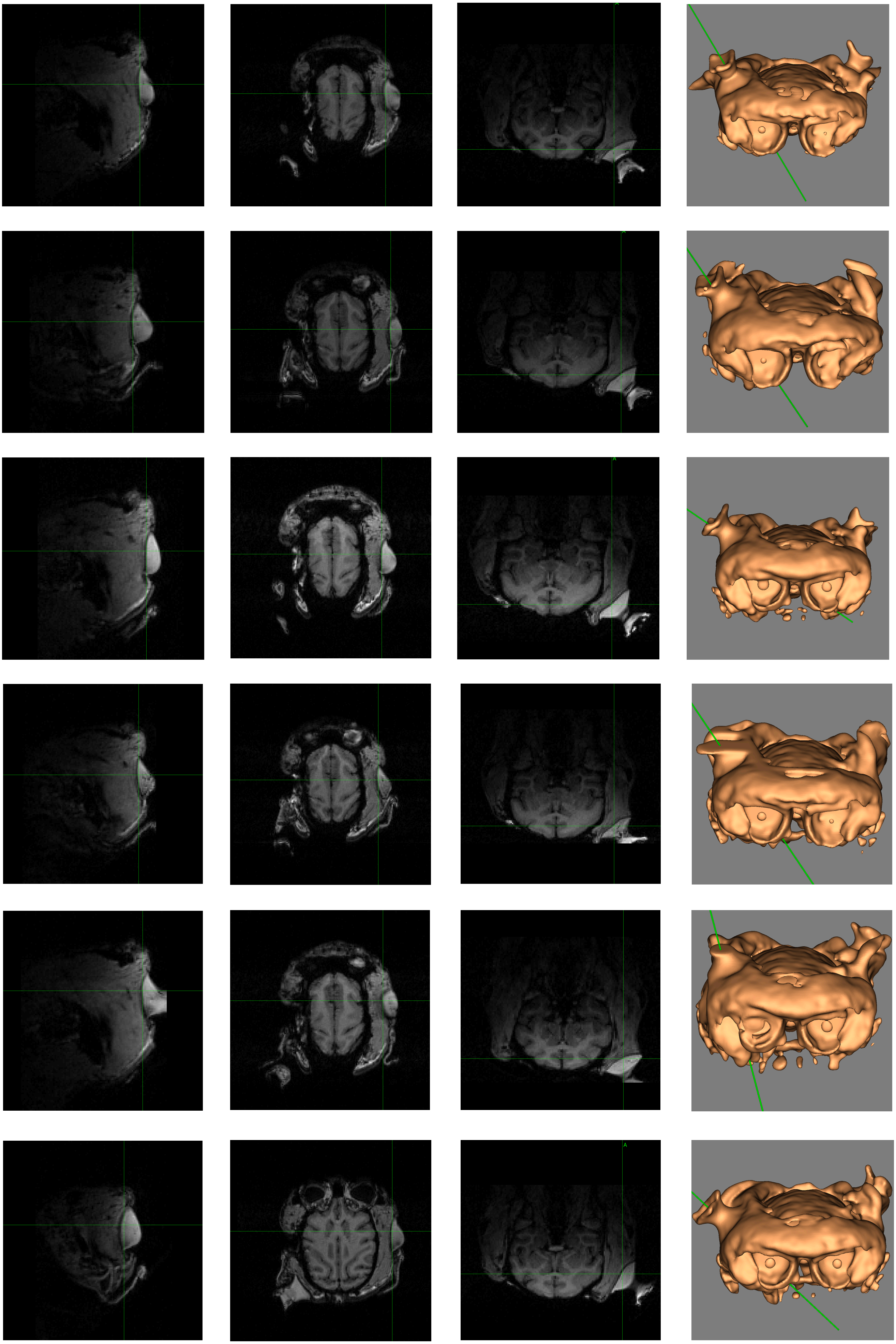

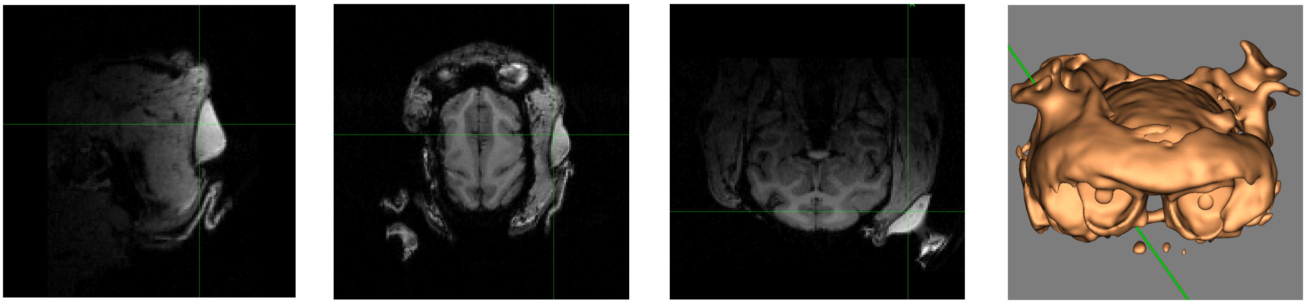

Cathodal electrode (prefrontal)

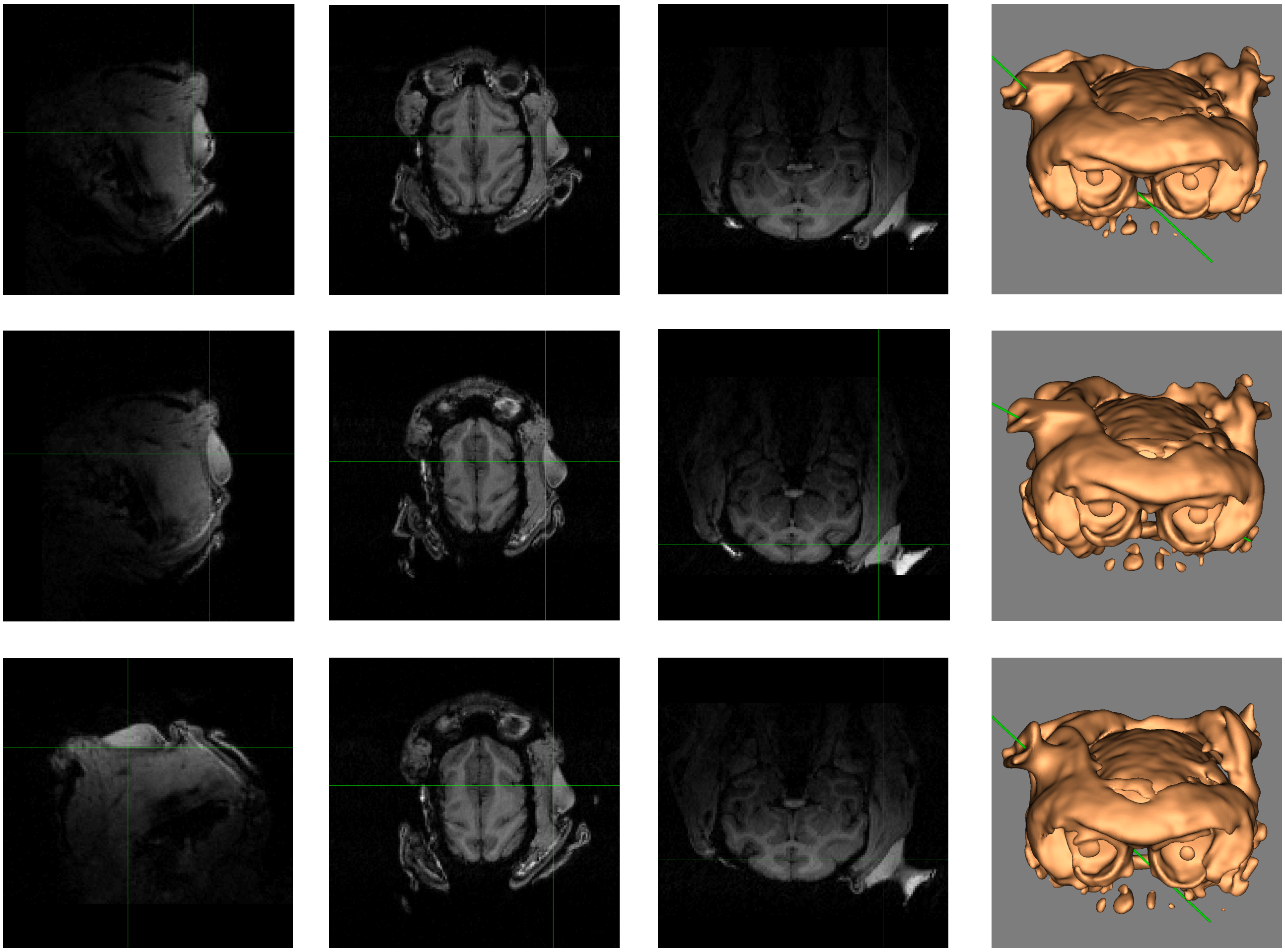

Cathodal electrode (occipital)

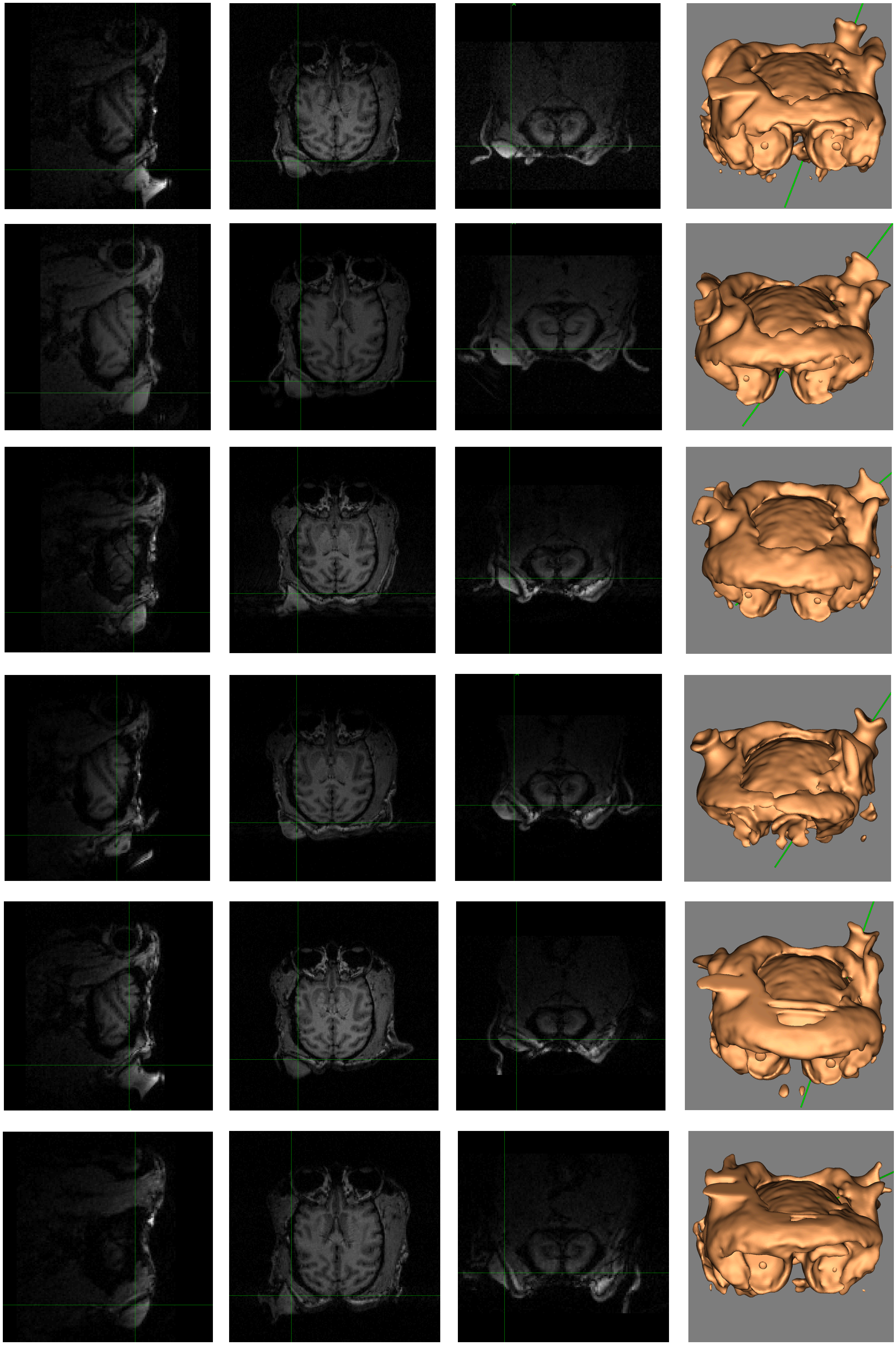

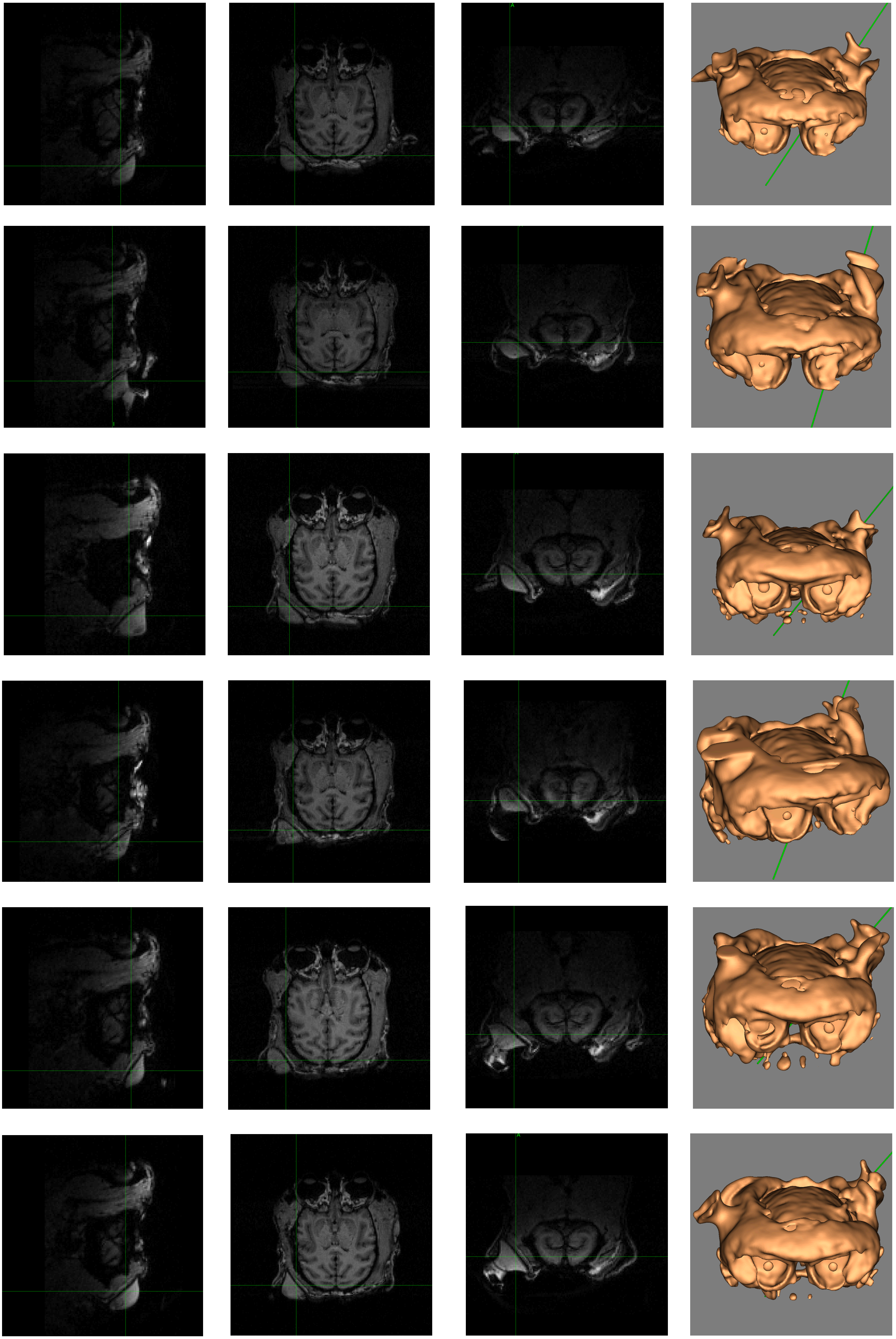

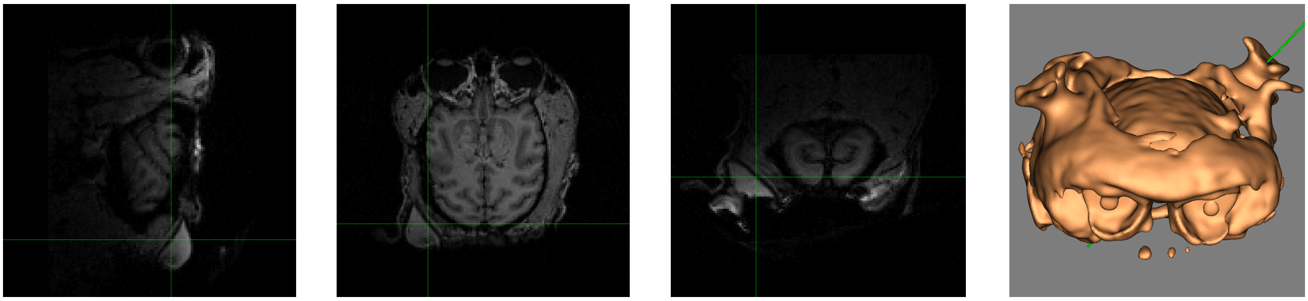

Anodal electrode (occipital)

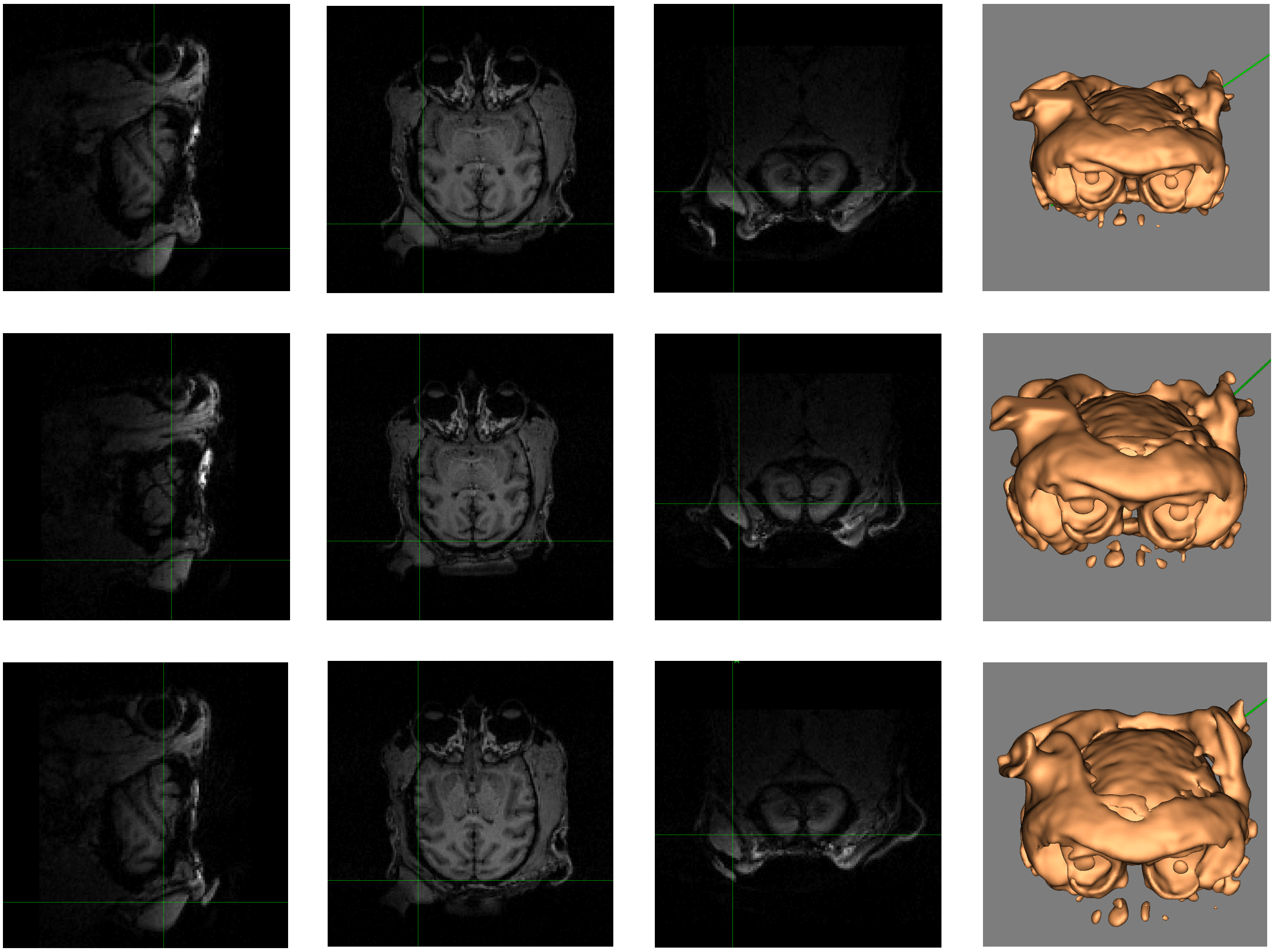

Monkey Y. (14 MRI sessions)

Anodal electrode (prefrontal)

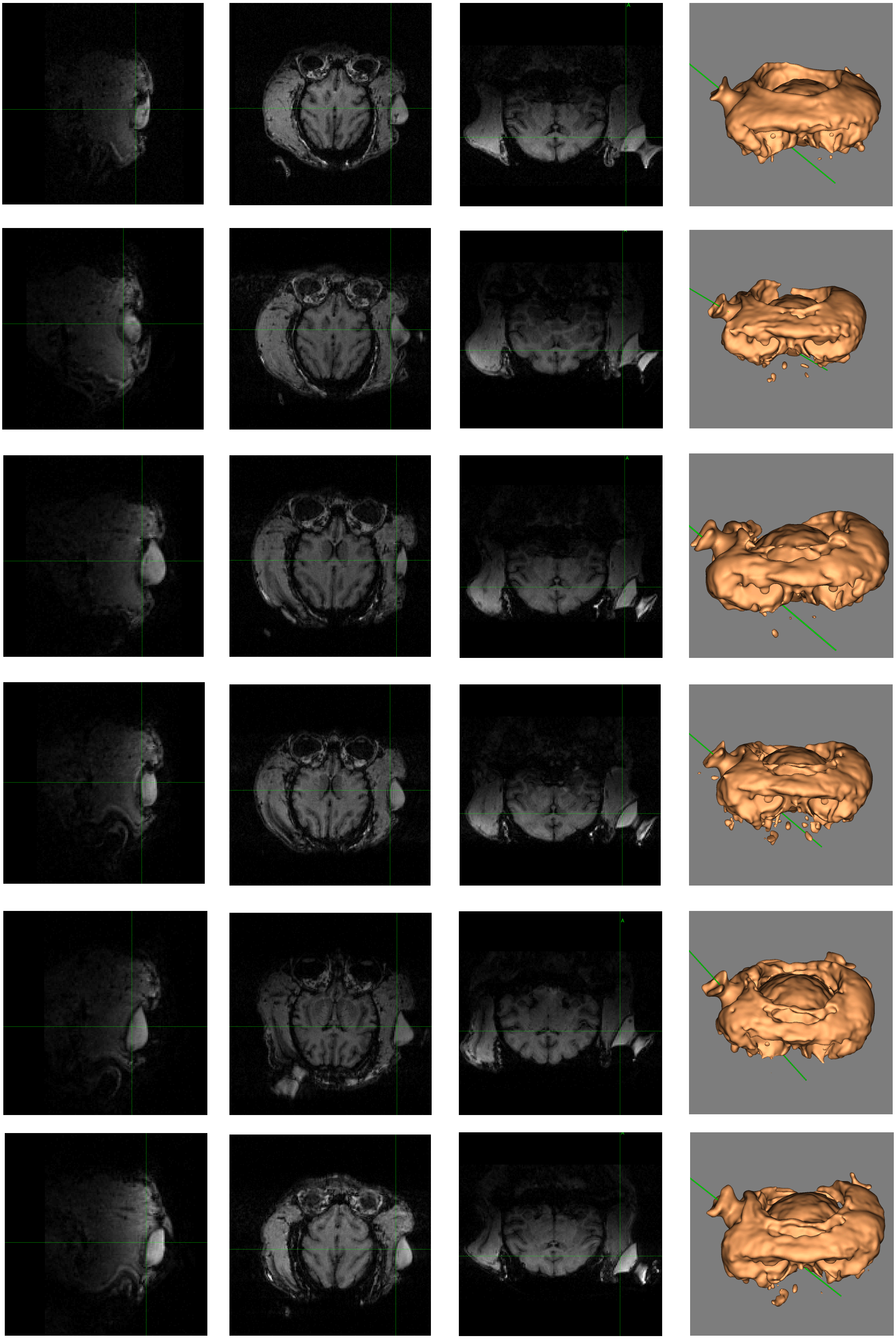

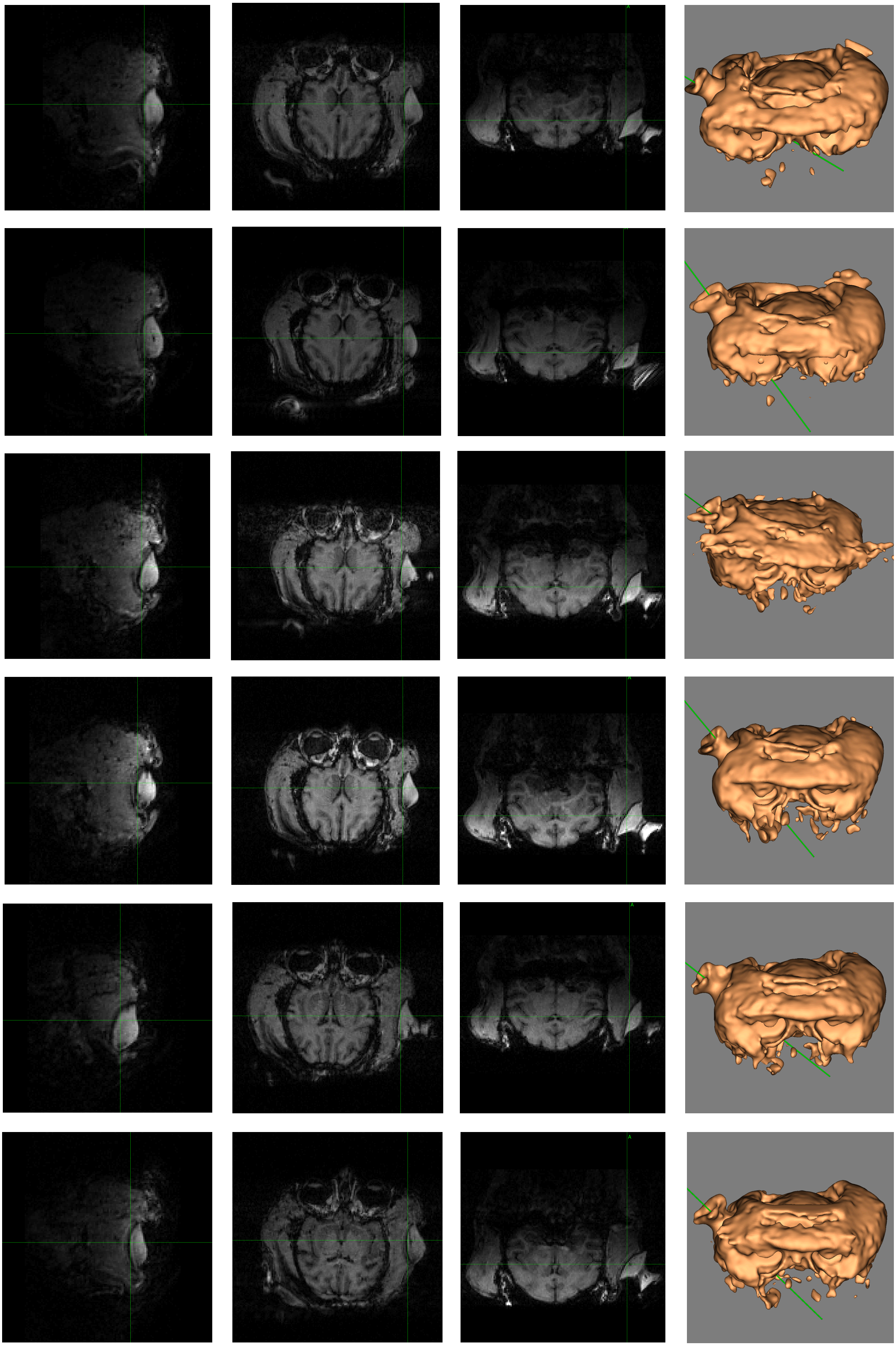

Cathodal electrode (prefrontal)

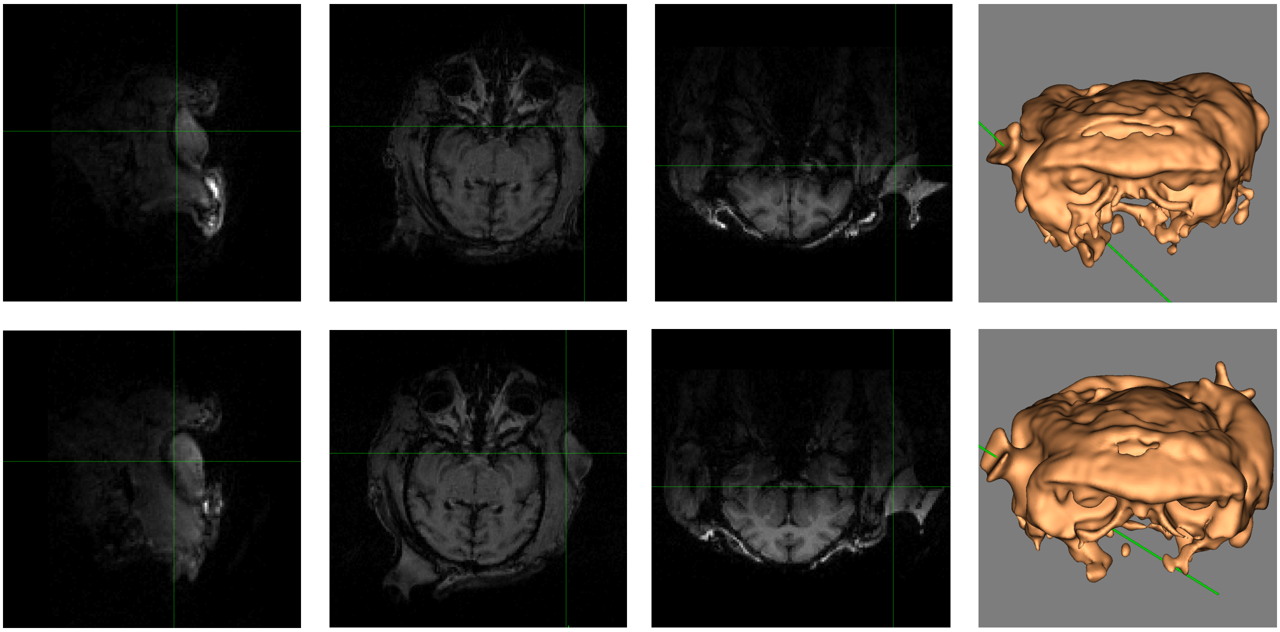

Cathodal electrode (occipital)

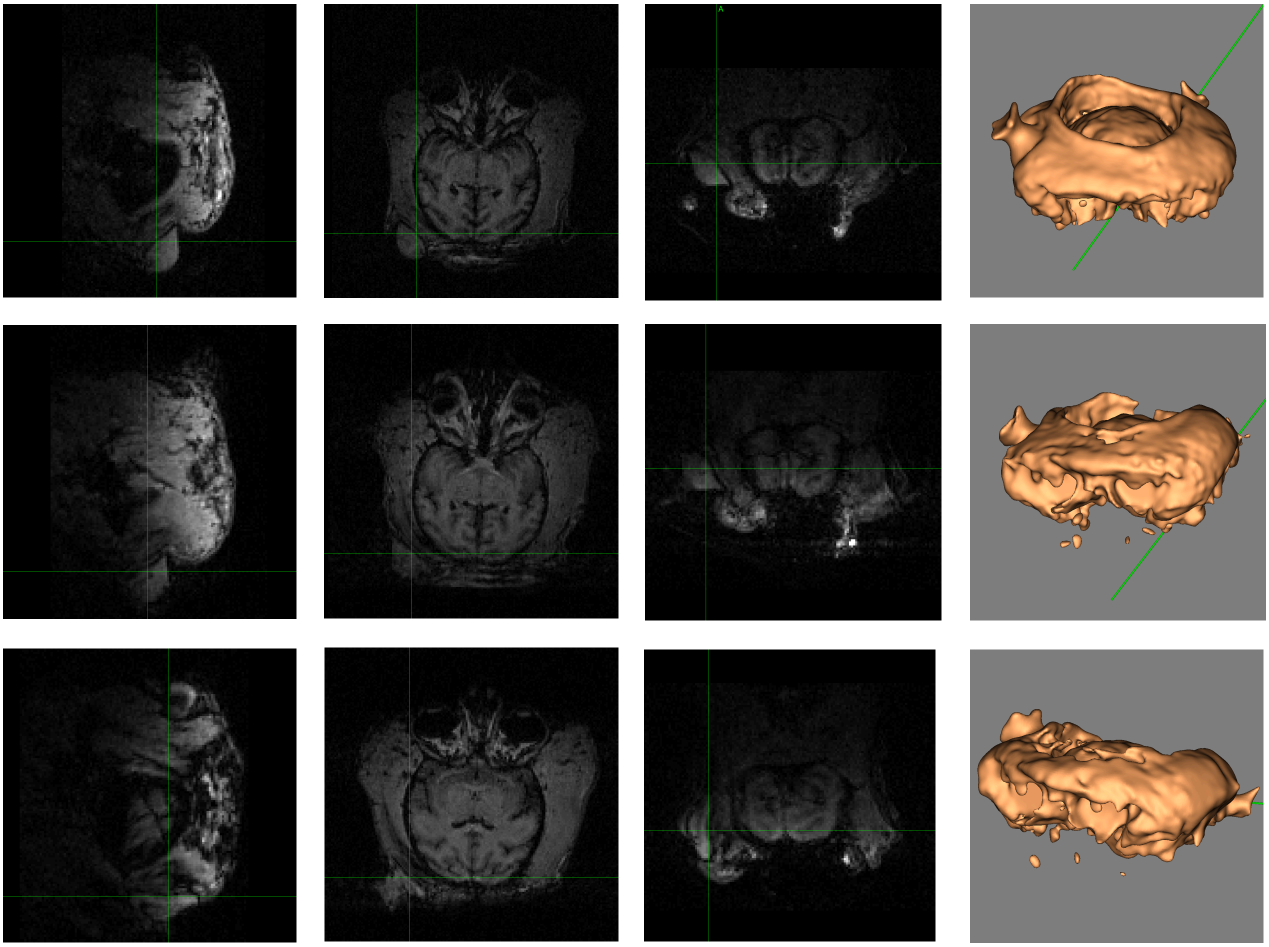

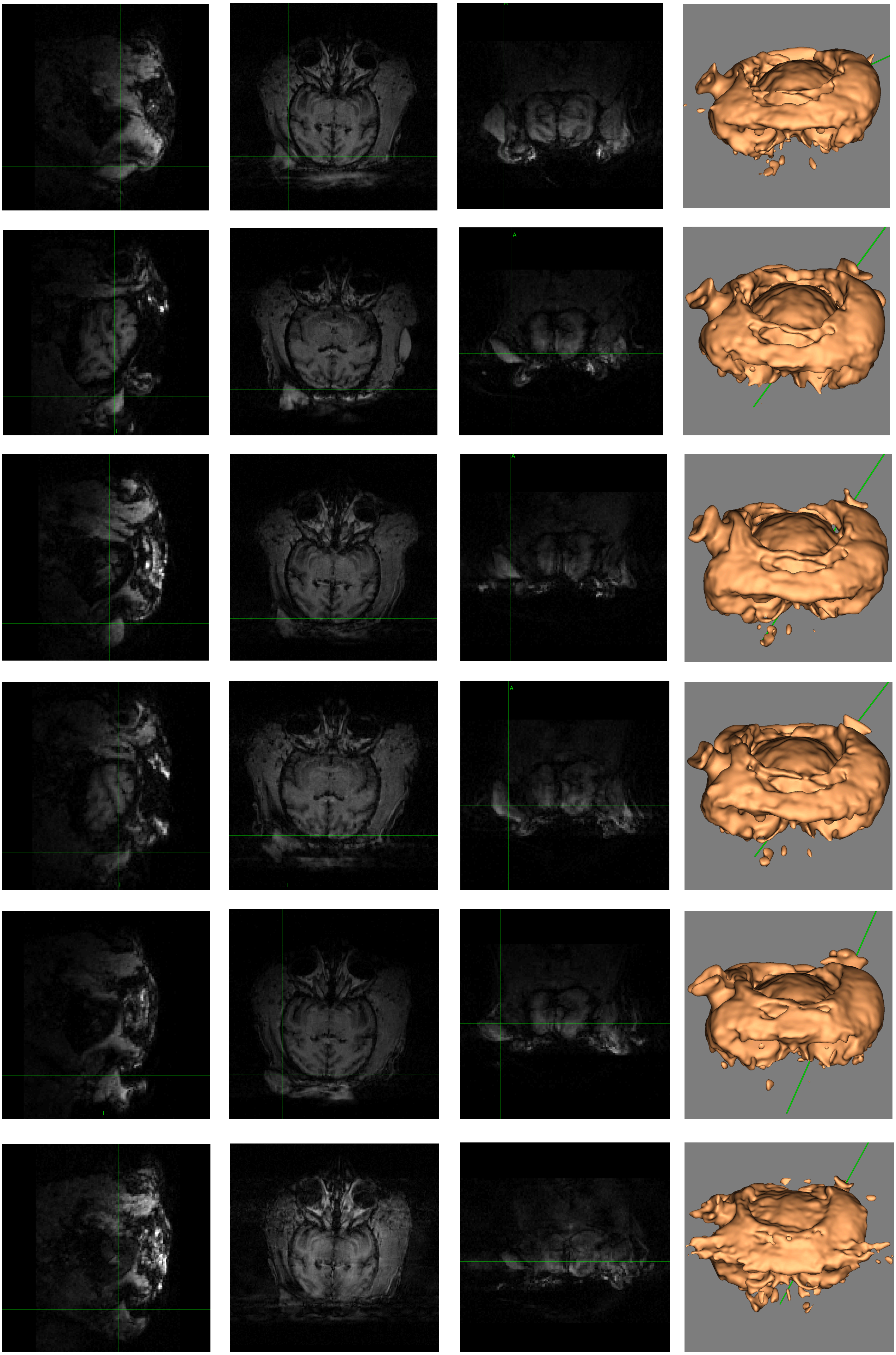

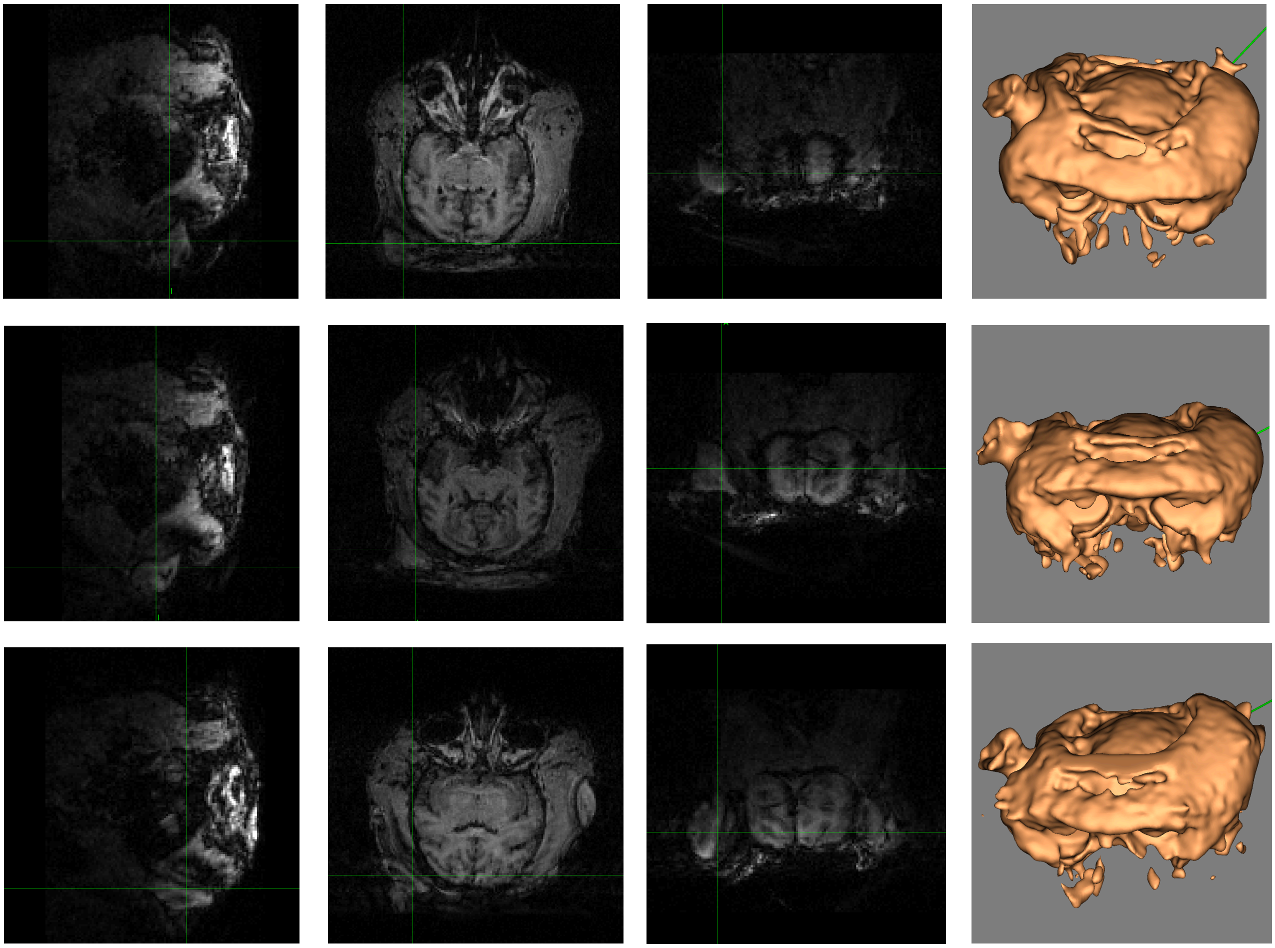

Anodal electrode (occipital)

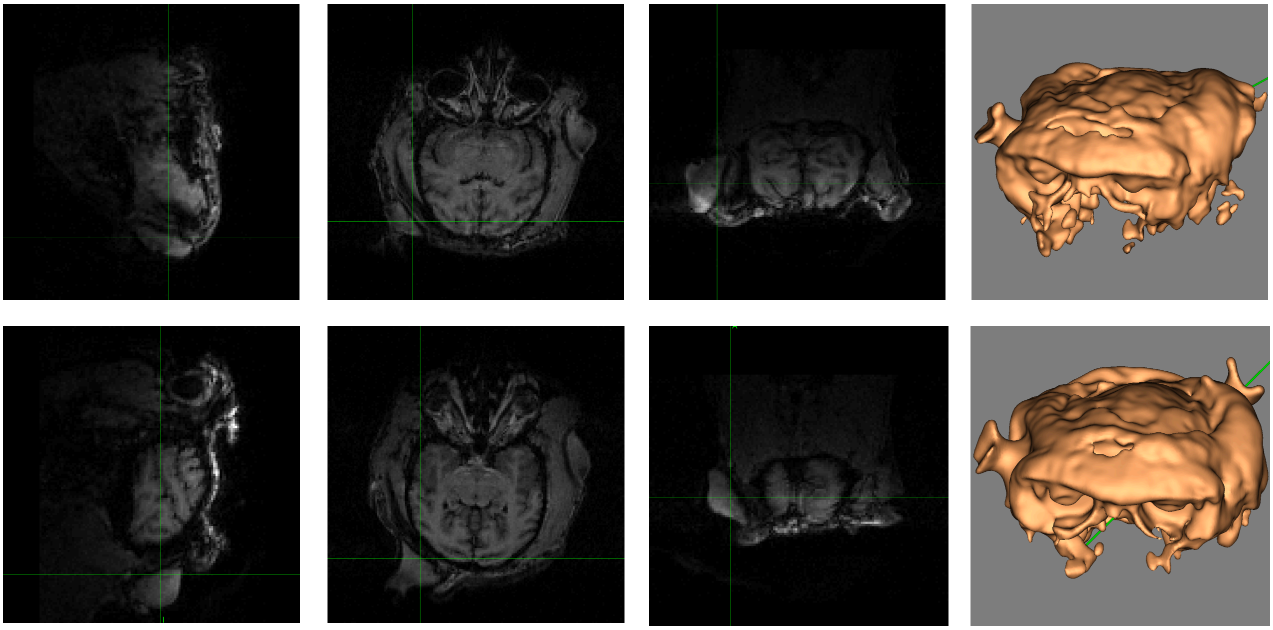

Supplement: Supplementary file 1. — Sagittal, coronal, and transverse MRI sections, and corresponding skin reconstruction images showing the position of the prefrontal and occipital electrodes on the monkey’s head for each MRI session (except for four sessions in which no anatomical scan was acquired). The two electrodes were accurately placed over the prefrontal cortex and the occipital cortex in a reproducible manner across sessions and between the two monkeys studied in each arousal state. In anesthesia experiments, the anodal electrode was placed over the dorsal prefrontal cortex, while the cathodal electrode was positioned over the parieto-occipital junction. In awake experiments, the prefrontal electrode was positioned over the dorsal prefrontal cortex/premotor cortex, while the occipital electrode was placed over the visual area 1. The position of the two electrodes differed slightly between the anesthetized and awake experiments due to different body positions (the prone position of the sedated monkeys prevented a more posterior position of the occipital electrode) and also due to the presence of a headpost on the head of the two monkeys in awake experiments (the monkeys we worked with in anesthesia experiments did not have a headpost). [file elife-101688-supp1.pdf]
