# Supplementary figures and images for: Transcranial direct current stimulation modulates primate brain dynamics across states of consciousness
Source: eLife. 2025 Oct 13;13:RP101688. doi: 10.7554/eLife.101688 (PMC12517689; doi:10.7554/eLife.101688)

**A**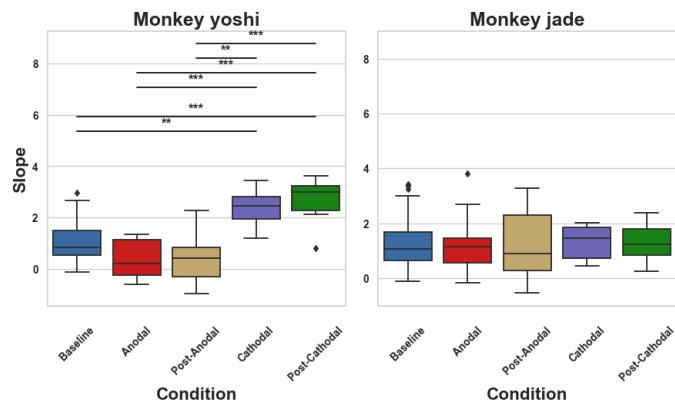**B**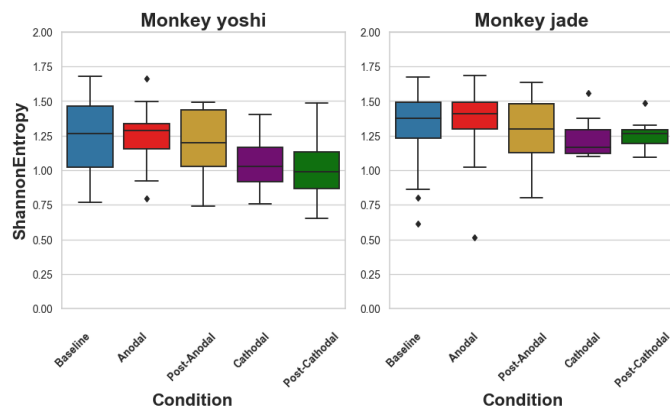**C**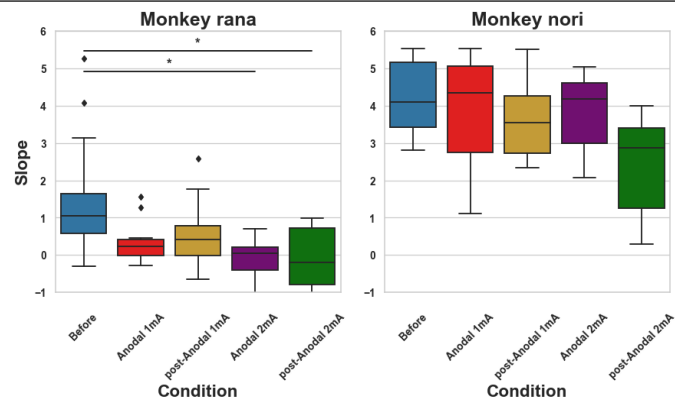**D**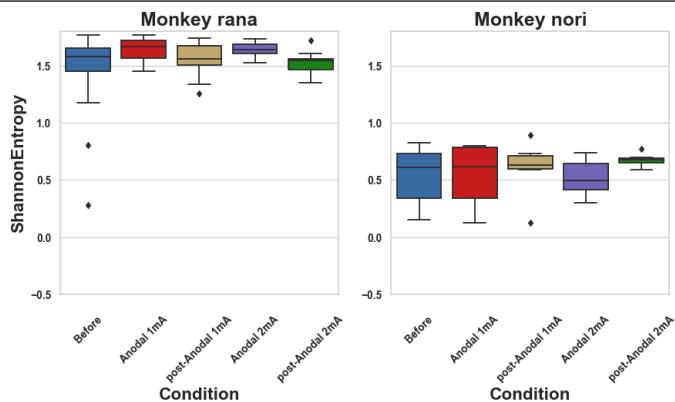

Supplement: Supplementary file 2. — (A) Slope analysis performed for both monkeys (Y. and J.) separately in the only awake conditions. (B) Shannon entropy analysis performed for both monkeys (Y. and J.) separately in the only awake conditions. (C) Slope analysis performed for both monkeys (Y. and J.) separately in the only anesthesia conditions. (D) Shannon entropy analysis performed for both monkeys (Y. and J.) separately in the only anesthesia conditions. [file elife-101688-supp2.pdf]
