# Supplementary material for: Transcranial direct current stimulation modulates primate brain dynamics across states of consciousness
Source: eLife. 2025 Oct 13;13:RP101688. doi: 10.7554/eLife.101688 (PMC12517689; doi:10.7554/eLife.101688)
Supplement: Supplementary file 3. — (A) Particular region-to-region functional links are depicted by their p-value significance when comparing pairs of conditions. (B) Modularity of average FC matrices shows no difference between conditions, (C) neither does it when computed for all the visited functional phase coherence states (for every time point, for all subjects), nor efficiency measure or density. [file elife-101688-supp3.pdf]

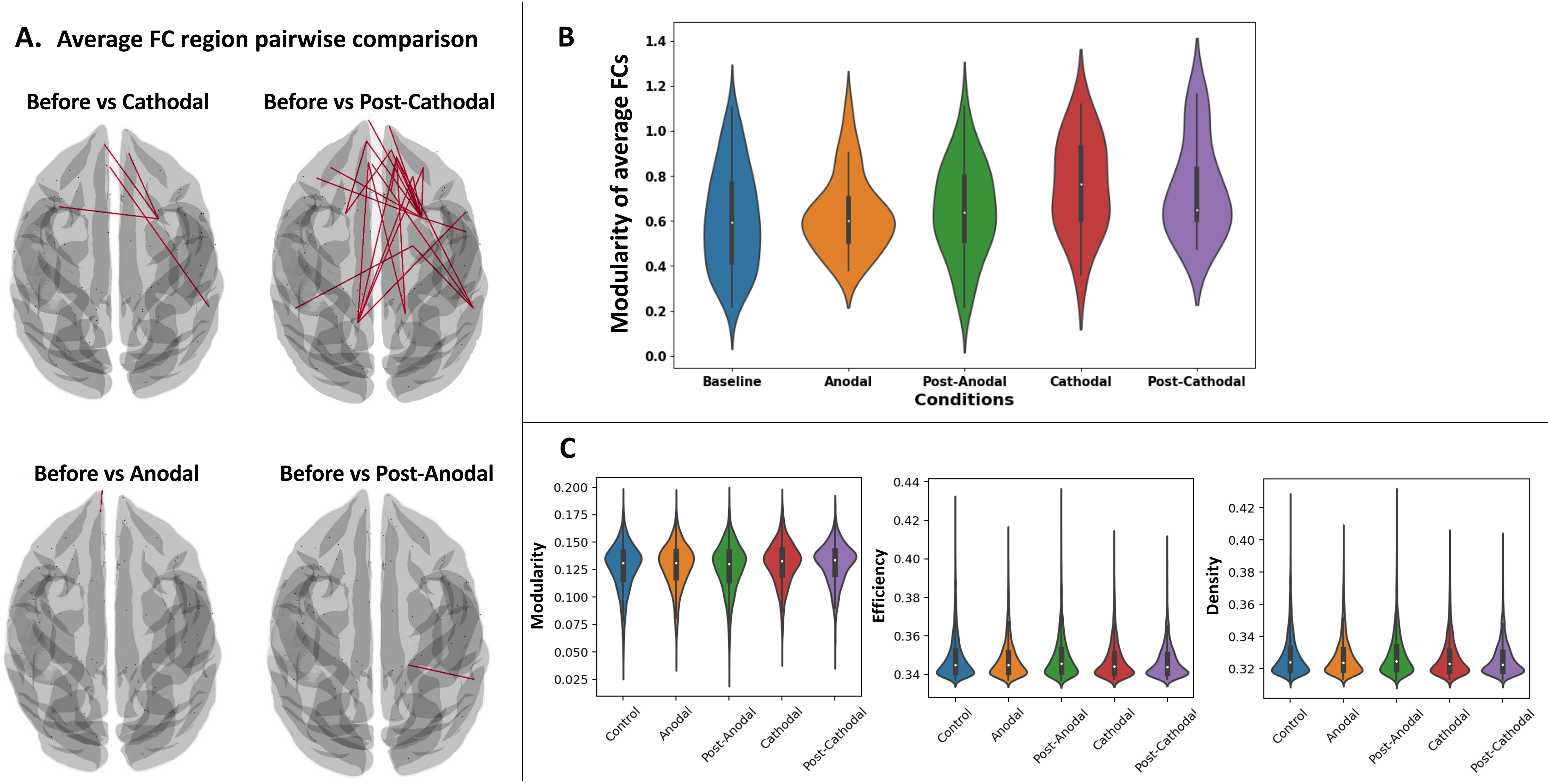

A. Average FC region pairwise comparison
Before vs Cathodal
Before vs Post-Cathodal
Before vs Anodal
Before vs Post-Anodal
B
Modularity of average FCs
1.4
1.2
1.0
0.8
0.6
0.4
0.2
0.0
Baseline
Anodal
Post-Anodal
Cathodal
Post-Cathodal
Conditions
C
Modularity
0.200
0.175
0.150
0.125
0.100
0.075
0.050
0.025
Control
Anodal
Post-Anodal
Cathodal
Post-Cathodal
Efficiency
0.44
0.42
0.40
0.38
0.36
0.34
Control
Anodal
Post-Anodal
Cathodal
Post-Cathodal
Density
0.42
0.40
0.38
0.36
0.34
0.32
Control
Anodal
Post-Anodal
Cathodal
Post-Cathodal
