# Supplementary material for: Transcranial direct current stimulation modulates primate brain dynamics across states of consciousness
Source: eLife. 2025 Oct 13;13:RP101688. doi: 10.7554/eLife.101688 (PMC12517689; doi:10.7554/eLife.101688)
Supplement: Supplementary file 4. — (A) Particular region-to-region functional links are depicted by their p-value significance when comparing pairs of conditions. Not a single functional connection was found to be statistically significant. (B) Modularity of average FC matrices shows no difference between conditions, (C) neither does it when computed for all the visited functional phase coherence states (for every time point, for all subjects), nor efficiency measure or density. [file elife-101688-supp4.pdf]

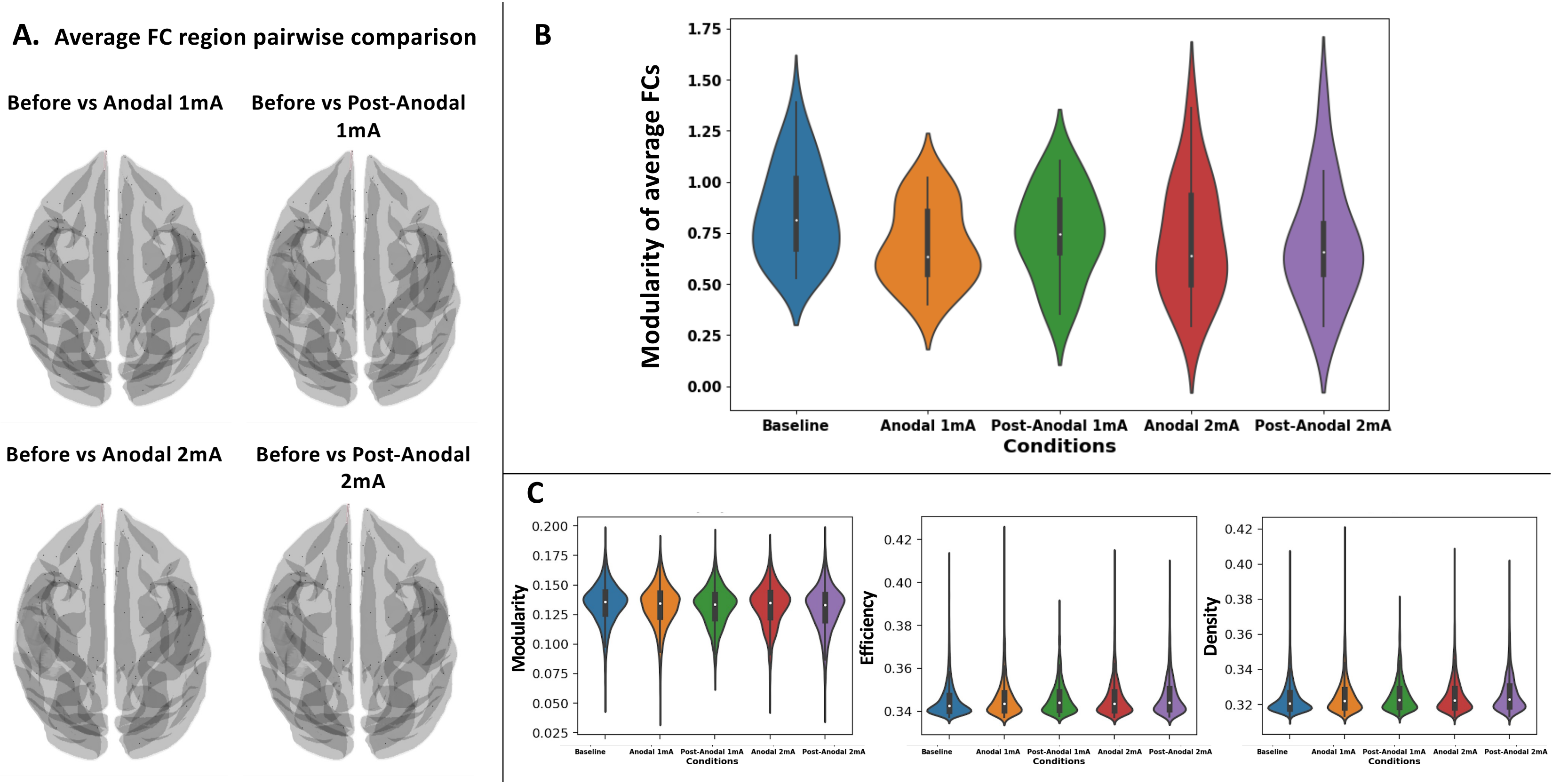

A. Average FC region pairwise comparison
Before vs Anodal 1mA
Before vs Post-Anodal 1mA
Before vs Anodal 2mA
Before vs Post-Anodal 2mA
B
Modularity of average FCs
1.75
1.50
1.25
1.00
0.75
0.50
0.25
0.00
Baseline
Anodal 1mA
Post-Anodal 1mA
Anodal 2mA
Post-Anodal 2mA
Conditions
C
Modularity
0.200
0.175
0.150
0.125
0.100
0.075
0.050
0.025
Efficiency
0.42
0.40
0.38
0.36
0.34
Density
0.42
0.40
0.38
0.36
0.34
0.32
Baseline
Anodal 1mA
Post-Anodal 1mA
Anodal 2mA
Post-Anodal 2mA
Conditions
